# Supplementary material for: Cost-effectiveness of QuantiFERON-TB Gold In-Tube versus tuberculin skin test for diagnosis and treatment of Latent Tuberculosis Infection in primary health care workers in Brazil
Source: PLoS One. 2019 Nov 14;14(11):e0225197. doi: 10.1371/journal.pone.0225197 (PMC6855475; doi:10.1371/journal.pone.0225197)
Supplement: Attachment — Submitted filename: Response to Reviewers PONE-D-19-15042_15OUT2019.docx [file pone.0225197.s002.docx]

PONE-D-19-15042

Cost-Effectiveness of QuantiFERON®-TB Gold In-Tube Versus Tuberculin Skin Test for Diagnosis and Treatment of Latent Tuberculosis Infection in Primary Health Care Workers in Brazil

Dear Editors,

The authors are grateful for the suggestions of the reviewers, who point out as very important for the manuscript improvement.

Please also be advised that changes and additions to version 2 text are marked as requested and an unmarked version of revised paper without tracked changes has been included (labeled 'Manuscript').

A summary of the changes made in response to the requests is provided below to facilitate the evaluation of the achievement.

Regards,

Jose Ueleres Braga

**Review Comments to the Author**

**Reviewer #1:**

This was an interesting and informative study comparing cost effectiveness of 5 strategies for diagnosis of latent TB in health care workers in Brazil. The evaluation was based on a hypothetical cohort of 10,000 HCW. Outcomes evaluated included number of individuals correctly classified and number of TB cases avoided. The conclusions of the study were that TST with a ≥10 mm cutoff was the most cost saving strategy for diagnosis of latent TB in this population.

Overall:

The text is a bit difficult to read with some issues with word order, etc that I believe are a byproduct of the translation to English. I would suggest having a native English speaker edit the manuscript if possible.

R.: Yes. We reviewed the entire manuscript with a native English speaker, looking to correct the issues identified.

Minor points:

Authors use both 10 mm and ≥10 mm throughout the manuscript. ≥10 mm should be used consistently.

R.: We agree. We replace the expression 10mm with ≥10mm when used to classify individuals by Mtb infection status.

Line 49: Suggest editing to say “preventing active disease” or “identification of latent and active disease in an effort to reduce transmission”

R.: The text of the manuscript was modified to comply with the suggestion made by the reviewer.

Lines 51-53: Suggest updating to more recent statistics.

R.: The text has been modified to include the latest tuberculosis statistics in Brazil and worldwide, with appropriate replacement in the reference section.

Lines 118-122: This text is repetitive with the figure legends and unnecessary.

R.: We agree with the reviewer's comment and the text has been rewritten to eliminate the pointed repetition.

Line 129: Please edit title of Figure 2 so that it is not identical to the title of Figure 1.

R.: We agree. The titles of figures 1 and 2 have been edited to clarify what is in place. Unnecessary repetitions when displayed in text were deleted.

373-375: The effectiveness of all strategies in regard to number of tuberculosis cases avoided is very similar. Is the difference between the strategies statistically significant? Mention should be made of the closeness of all strategies for this measure of outcome effectiveness.

R.: We agree. The manuscript was revised to highlight the closeness of the effectiveness values. No statistical tests were applied to compare these figures

Line 440: “…the change in the most cost-effective strategy was a result of the substantial increase in costs.” Suggest describing which costs were increased.

R.: The text was rewritten to include the suggestion of the reviewer.

Line 494: define STF

R.: We apologize for the typo. The correct acronym is QTF

Table 1: I do not understand the calculation for “Cost of Complete LTBI Treatment(6months)”, “Cost of Partial LTBI Treatment (3 months)” or “Cost of Active TB Treatment”. Why do line items for the drug(s) used for treatment have “(month)” beside them? Does this mean that this is the cost for one month? If so, the calculations for the total cost of treatment are incorrect since the values have only been included once. If these values actually represent the cost of drug for the entire duration of treatment why do they have “(month)” beside them?

R.: We appreciate the remark. The values in table 1 correspond to the total cost of treatment. The term “month” has been removed to avoid misunderstanding.

Discussion:

There is no discussion of the comparable results for all the tests for number of cases avoided.

R.: The discussion section has been rewritten and this aspect has been covered.

Other Suggestions:

• I think that the addition of number-needed-to treat (the number of people treated for LTBI to prevent an active TB case) for each strategy would be an interesting and valuable addition to the study.

R.: We welcome the suggestion, but understand that the epidemiological measures used are considered appropriate for this cost-effectiveness study.

• Authors mention that adherence to LTBI treatment by HCW is low due to the known false-positivity rate of TST. Is the adherence rate to LTBI treatment for individuals diagnosed with QFT known or can it be realistically estimated? Would it be possible to do a cost calculation for TST vs QFT using different adherence rates for each test? This could be really important since the rate of adherence to LTBI treatment was one of the most impactful variables in the cost effectiveness calculations and would also likely impact number of active TB cases avoided.

R.: There were no published studies on adherence to LTBI treatment after QTF screening at the time of development of the current study. A literature search to answer the reviewer found few updated studies addressing treatment adherence rate after QTF testing, and their values are compatible with the parameters assumed in this study. A bivariate sensitivity analysis was performed considering different adherence rates after diagnosis when each test was used, and its result did not indicate a change in the conclusion, in favor of the TST strategy with 10mm cut-off. There were insignificant changes in the number of active TB cases avoided by applying different LTBI treatment adherence rates according to the diagnostic strategy employed.

**Reviewer #2:**

The paper present interesting data investigating the cost effectiveness of QuantiFERON®-TB Gold In-Tube and Tuberculin Skin Test for Diagnosis and Treatment of Latent Tuberculosis Infection in Primary Health Care Workers in Brazil. The method section is well explained, however the result and discussion sections need to be restructured in a better way.

The tables in the results section (especially table 4) are too large, they need to be presented in a more efficient way. a summarizing table can be used and the rest of the data can be attached to the paper as supporting information.

The manuscript need to be English edited in a very detailed way. Many of the sentences are too long and contain grammar and formulation mistakes.

R.: Thanks for the suggestion, we've reformatted tables to make your look cleaner. However, we believe it would be relevant for the reader to keep part of the information present in the manuscript in its original format. Following the suggestions we also rewrote the result and discussion sections to make the content clearer. Finally, the manuscript was reviewed by a native American to minimize errors with the use of the English language.

Some suggestions and comments below:

Line 59: add more recent statistics

R.: The text has been modified to include the latest tuberculosis statistics in Brazil and worldwide, with appropriate replacement in the reference section.

Line 65: is considered as a priority by WHO in controlling the disease ??

R.: Yes. WHO considers the identification of LTBI and its treatment in priority groups as an important strategy for disease control, and this view holds in the latest report available (2018).

Line 138: do you mean true positives and true negatives?

R.: Yes. The text has been modified to make it clear what was being said.

Line 181: cut-off

R.: We accept the suggestion and change the expression cutoff for cut-off throughout the text.

184-185: data based on...

R.: We apologize. The text has been corrected.

351-352: column 2 in table 2, is it the total cost, if so mention it

R.: We apologize. The text has been corrected.

453: what do you mean by study contacts?

R.: We revised the text to make it clearer..

471: are you comparing the study results to other studies performed outside of Brazil? if so, find a good way to make the transition between this paragraph and the previous one

R.: We thank the suggestion and rewrite the text, leaving separated the studies with concordant and discordant results from the evidenced in the present research.

549-550: this paragraph is out of context, you will need to develop it further and find a way to link it to the previous paragraph (or you can delete it)

R.: Thanks. We accept your suggestion.

While revising your submission, please upload your figure files to the Preflight Analysis and Conversion Engine (PACE) digital diagnostic tool, <https://pacev2.apexcovantage.com/>. PACE helps ensure that figures meet PLOS requirements. To use PACE, you must first register as a user. Registration is free. Then, login and navigate to the UPLOAD tab, where you will find detailed instructions on how to use the tool. If you encounter any issues or have any questions when using PACE, please email us at [figures@plos.org](mailto:figures@plos.org). Please note that Supporting Information files do not need this step.
